# Long-read sequencing for brain tumors

**DOI:** 10.3389/fonc.2024.1395985

**Published:** 2024-06-10

**Authors:** William J. Shelton, Sara Zandpazandi, J Stephen Nix, Murat Gokden, Michael Bauer, Katie Rose Ryan, Christopher P. Wardell, Olena Morozova Vaske, Analiz Rodriguez

**Affiliations:** ^1^ Department of Neurosurgery, College of Medicine, University of Arkansas for Medical Sciences, Little Rock, AR, United States; ^2^ Department of Neurosurgery, Medical University of South Carolina, Charleston, SC, United States; ^3^ Department of Pathology, University of Arkansas for Medical Sciences, Little Rock, AR, United States; ^4^ Department of Biomedical Informatics, University of Arkansas for Medical Sciences, Little Rock, AR, United States; ^5^ Department of Biochemistry and Molecular Biology, University of Arkansas for Medical Sciences, Little Rock, AR, United States; ^6^ Department of Molecular, Cell and Developmental Biology, University of California Santa Cruz, Santa Cruz, CA, United States

**Keywords:** brain tumors, third generation sequencing, long-read sequencing, molecular diagnostics, liquid biopsy

## Abstract

Brain tumors and genomics have a long-standing history given that glioblastoma was the first cancer studied by the cancer genome atlas. The numerous and continuous advances through the decades in sequencing technologies have aided in the advanced molecular characterization of brain tumors for diagnosis, prognosis, and treatment. Since the implementation of molecular biomarkers by the WHO CNS in 2016, the genomics of brain tumors has been integrated into diagnostic criteria. Long-read sequencing, also known as third generation sequencing, is an emerging technique that allows for the sequencing of longer DNA segments leading to improved detection of structural variants and epigenetics. These capabilities are opening a way for better characterization of brain tumors. Here, we present a comprehensive summary of the state of the art of third-generation sequencing in the application for brain tumor diagnosis, prognosis, and treatment. We discuss the advantages and potential new implementations of long-read sequencing into clinical paradigms for neuro-oncology patients.

## 1 Introduction

The World Health Organization (WHO) classification of central nervous system (CNS) tumors now requires the integration of histopathology and molecular genetics demonstrating the need for molecular characterization clinically (1). With the advent of precision medicine in oncology, wherein targetable mutations are identified for therapies, the application of next-generation sequencing will continue to expand. Specifically in the field of neuro-oncology, the incidence of brain tumors continues to increase necessitating the integration of novel sequencing methods into clinical paradigms (2, 3). One of the promising new applications is the use of third-generation sequencing or long-read sequencing (LRS). In this review, we describe the new opportunities for LRS to be of utility for diagnosis, prognosis, and treatment strategy development for CNS tumors.

## 2 Brain tumors

The prevalence of brain tumors has been increasing over the decades (2, 4). Furthermore, they have been associated with higher prevalence and mortality rates in countries with a high human development index (HDI), such as the United States (5). An epidemiological overview provided by The Central Brain Tumor Registry of the United States (CBTRUS) from 2015-2019 showed an “average annual age-adjusted incidence rate (AAAIR) of all malignant and non-malignant brain and other CNS tumors” of 24.71 per 100,000 (6). Malignant brain tumors have a grim prognosis, with only one-third of individuals surviving 5 years after their initial diagnosis (3).

The World Health Organization (WHO) has been the worldwide standard reference for CNS tumors classification since they published their first guideline more than 40 years ago (7). A classification system was established by the WHO to group tumors based on their pathologic characteristics, clinical presentation, and patient demographic similarities (7). This classification not only enhanced clinical practice by providing physicians and patients with a better understanding of prognosis and treatment options but also laid the groundwork for researchers to develop methods aimed at improving disease prognosis. Initially, CNS tumors were classified based on histopathological diagnosis of tissue samples. However, the classification does not always correlate with the clinical outcome of patients and can sometimes be misleading (8). For instance, histopathological diagnosis is known to present “intra- and inter-observer variability”, leading to variations in the grading of disease severity (9, 10). There is a lack of clinical prognosis and correlation with histological features in certain types of tumors, such as in pediatric posterior fossa ependymomas (11) or in diffuse gliomas (12). These factors collectively contribute to a reduced likelihood of achieving accurate diagnoses (8, 9, 13–15). As a result, updates in the classification system led to the incorporation of molecular markers for the first time in 2016 (16, 17). The most recent WHO guidelines, known as CNS 5, were released in 2021 and have further broadened the requirements for genomic analysis of tumors (18, 19).

## 3 Genetic insights of brain tumors

Advances in the understanding of cancer genomics have significantly improved over the last decade, primarily attributed to the genetic profiling of tumors (20). The recognition of different genetic alterations and their associated pathways not only has allowed for a better grouping based on similarities and responses to treatment but has also provided targetable genetic alterations for molecular therapies (21). A spectrum of genetic alterations are known to be key factors in the development of tumors such as glioblastoma (GBM), one of the most studied and deadly CNS tumors (22, 23). Despite the numerous and continuous efforts to approach this disease (24), the prognosis remains poor, with the median survival only improved to approximately 15 months with the introduction of the Stupp protocol in 2005 (radiotherapy plus concomitant chemotherapy with the alkylating drug, temozolomide) (25). Genomic analysis of CNS tumors has allowed for an understanding of the multiple drivers that promote molecular alterations, such as genetic and epigenetic modifications, activation of cancer stem cell pathways, and the tumor microenvironment (22, 26). The addition of diagnostic molecular biomarkers is fundamental for the integrated diagnosis of these tumors. For example, for the diagnosis of a diffuse glioma (according to the WHO CNS 5) it is required to know the status of the isocitrate dehydrogenase (IDH) gene mutation for further subclassification (18). While traditional molecular assays (e.g. immunochemistry) have been used to identify commonly known mutations of characteristic genes, such as IDH1 R132H, or even nucleic acid-based technologies (e.g. Sanger sequencing) to determine phenotypic variations of these mutations (e.g. IDH1 R132S), the validity and consensus on these techniques are still insufficient (18, 27). The development of novel, more cost-effective, and rapid technologies that could comprehensively address cancer diagnosis by providing a complete genomic analysis through the simultaneous screening of multiple genetic biomarkers became imperative. Consequently, owing to the foundational work laid by the Human Genome Project, third generation sequencing (TGS), also known as LRS, emerged. Given the emerging need for robust genetic and epigenetic characterization of brain tumors for clinical decision-making, LRS has many emerging applications in neuro-oncology.

## 4 DNA sequencing techniques

### 4.1 First and second-generation sequencing technologies

Sequencing determines the precise order of nucleic acids in the deoxyribonucleic acid (DNA). DNA sequencing was properly introduced in 1977 with the development of Frederick Sanger’s ‘chain termination’ technique (28). Although previous methods for DNA sequencing existed, they were time-consuming and highly expensive (29). Sanger’s method employed radioactively or fluorescently labeled dideoxynucleotides (ddNTP) in four parallel DNA polymerization reactions, resulting in random incorporation into the DNA strands and termination of the reaction. Subsequently, by utilizing a polyacrylamide gel, the sequence would be read by looking at the migration of DNA fragments (29). Modern Sanger sequencing uses capillary-based electrophoresis and automated DNA sequencing machines (29). While Sanger’s method was once considered the gold standard for DNA sequencing, it had significant drawbacks, primarily being expensive and time-consuming, especially considering the limited number of sequences in a single experiment (800-1000 base pairs) (28, 30–32). In 2005, a revolutionary technology called ‘next generation sequencing’, also referred to as ‘second generation sequencing’, was introduced. These technologies led to a substantial increase in sequencing data output due to various technological innovations that enabled the sequencing of a much larger quantity of DNA molecules in a more time- and cost-effective manner (30). The comparison between this technique and the traditional method is outstanding, as second-generation sequencing can sequence the genome of a small organism in just one day (31). This technique differs from Sanger sequencing as it allows for the continuous incorporation of enzymatic nucleotides, enabling continuous data acquisition (unlike Sanger’s technique). It also allows a large number of templates to run simultaneously, as it employs an array-based sequencing method in which DNA templates are compacted into a two-dimensional surface. This significantly reduces the costs of DNA sequencing, as a single reagent volume is needed per experiment (33–35). Furthermore, conventional sequencing is limited by the time-consuming E.coli transformation and colony picking as initial steps, while NGS relies on *in vitro* library construction with subsequent clonal amplification (34).

### 4.2 Third generation sequencing technologies

Despite the various improvements in this technique over the years, there are some important limitations. The need for template amplification in NGS technologies is not only time-consuming but also prone to PCR errors, particularly in regions with high GC content (36). Furthermore, artifactual mutations (e.g. DNA oxidation) can occur during sample preparation which can impact the downstream data analysis (37). Additionally, although NGS technologies offered a massive throughput, they still had limited read lengths (i.e. less than 200bp) which has shown to be a major limiting factor in the highly repetitive human genome (38). All of these factors led to the creation of new technologies that could combine the high throughput of NGS with longer read lengths than Sanger sequencing, all while being more affordable, rapid, and capable of delivering higher-quality results (39). This need was met with the introduction of the first LRS technology in 2011 by Pacific Biosciences (PacBio) and, subsequently, in 2014 by Oxford Nanopore Technologies (ONT) (36). Both technologies not only addressed the shortcomings of previous techniques by sequencing single molecules in real-time and offering a larger capacity but also expanded the possibilities of genomic research. One of the main advantages of these technologies is the possibility of producing long reads (between 10 kilobase to 15 kilobase) from a single DNA molecule (36). Characteristically, neither sequencing nor library preparation require PCR amplification, which presents as an enormous advantage as this lowers the cost, time, and related bias of PCR procedures (40) The costs required to cover each sequencing run are somewhat similar when comparing LRS devices with NGS. The sequencing cost per gigabase of PacBio RS and ONT are around $43-$86 and $21-42$ US dollars, respectively, while the cost per gigabase of Illumina is around $50-63$ US dollars (36, 41). Although NGS has been a reference technique for more than a decade, expanding the view of medical genetics with its high throughput and low-cost technique (42), LRS technologies have been acquiring more relevance for their growing potential in the application of improved genomic studies.

#### 4.2.1 Single-molecule real-time sequencing

Single-molecule real-time sequencing (SMRT) relies on a DNA polymerase immobilized in a well on a silicon chip. Two adaptors (called SMRTbells) are ligated to each end of the desired genomic sample to be sequenced. By binding a sequencing primer to the SMRTbell template, a complex is formed, which includes the ligation of the DNA polymerase, resulting in the creation of a circular double-stranded DNA molecule (43, 44). During the elongation of the new strand, phosphate labeled deoxynucleotides triphosphates (dNTPs) emit light signals, which are then detected and translated into a nucleotide sequence, commonly known as “base calling”. In each well, the DNA strand can undergo multiple rounds of elongation by the DNA polymerase until it stops, significantly reducing the error rates. After this process is done, a consensus sequence is generated for base calling. This technology has been proven invaluable for a wide range of genomic studies (45), as it can accurately identify up to 50kb of DNA molecules (46).

#### 4.2.2 Nanopore sequencing

On the other hand, nanopore sequencing is a single-molecule real-time sequencing technology that utilizes special channels or ‘pores’ through which single strands of DNA flow. These pores are separated by a membrane, creating compartments filled with ionic solutions (36). An adapter is ligated to the DNA, forming DNA-protein complexes. A polymerase or helicase enzyme is then added to facilitate the movement of DNA through the pores, aided by an ion transmembrane current. As the single stranded DNA passes through the pore, it causes disruptions in an ionic current, which is detected by sensors. This information is used for real-time base calling, and the technology is capable of producing extremely long sequencing reads, typically up to 30,000 base pairs but can be used for up to 1 million base pairs (47, 48). One of the major advantages of this technology is the ability to generate a large amount of data rapidly with high accuracy, making it well-suited for analyzing complex structural variants such as inversions, deletions, or translocations (49).

### 4.3 LRS applications

Different reviews, such as the one conducted by Mantere et al., have demonstrated the utility of LRS technologies by identifying novel elements of genomic alterations in known diseases (50–52). LRS has been employed to detect and map novel structural variants (52–57), sequence repetitive genomic regions (58–63), solve haplotype phasing (64–67), and discriminate pseudogenes (68–70). The versatility of LRS makes this technology invaluable for a range of genetic studies as this platform can be of significant utility in the creation of high-resolution genomic assemblies due to its long-read mechanism, which can accurately characterize a genome. The popularly used human genome reference (GRCh38) is a representation of the different existent haplotypes in the human being, but the telomere-to-telomere consortium utilized LRS for the development of the T2T-CHM13 reference genome which includes the complete genome (71). However, it is important to note that this genome may not fully capture the genetic diversity of the entire human population, as the data may be skewed towards the European population (72). Moreover, the pangenome, which contains genome assemblies from a diverse population was released in 2023 (73).The application of LRS in genomic assembly has addressed some of the existing gaps in the current reference genome (74, 75). LRS can be applied to other organisms, having the capability of showing the entire genome of a small organism within a single read (76). Further applications of this technology encompass targeted sequencing, transcriptomics, epigenetics, and a wide array of clinical applications including disease diagnosis, prognosis, and personalized medicine, which is particularly relevant for this review in the context of CNS cancer.

## 5 LRS in cancer

### 5.1 Genomics

Long read sequencing is valuable tool for studying the complexity of cancer genomes; characterized by multiple genetic and epigenetic alterations. Throughout the evolution of tumorigenesis, a tumor acquires and accumulates a wide variety of aberrations that promote certain characteristics for survival (77). These cancer mutations vary, presenting as simple substitutions, short insertions or deletions, and can also include more complex alterations such as gene fusions or chromosomal rearrangements, among others (77–79). The use of long read sequencing in a clinical setting could increase the detection of subclonal mutations, alternative splicing events and even characterize different isoforms of mRNA expression (80, 81). To demonstrate the utility of LRS in clinical scenarios, a study conducted by Watson et al. involved genetic analysis for the characterization of Meckel-Gruber syndrome, a lethal genetic disorder, in three fetuses. With the use of long-read sequencing, they were able to identify four missense variants arranged in a *trans* position of the TMEM231 gene. This was not possible to identify with short-read sequencing (82). The adaptability of this technology is based on the ability to sequence long genomic fragments, which is extremely useful for reading problematic regions, such as the highly repetitive ones that can be found in structural variants (SVs)-a key genetic alteration in oncogenesis (83). For instance, a recent study done by Xu et al. employed LRS for the first time in 21 colorectal cancer samples to investigate SVs. This study found that SVs were present in almost twice the number compared to previous studies using NGS. Furthermore, the use of this technology helped in the identification of a novel gene fusion in CRC, demonstrating the high advantages of LRS in cancer research (84).

One of the mechanisms of carcinogenesis involves gene fusions, which typically result from chromosomal arrangements (80, 85). This process gives rise to chimeric proteins that drive clonal expansion of abnormal cells, thus triggering oncogenesis. In brain cancer, a wide variety of gene fusions have been studied and are known to be involved in cancer pathways. A systematic review by You et al. identified 15 known gene fusions in adult-type diffuse gliomas, highlighting the significance of this genetic mechanism in CNS cancer (86). New techniques, such as LRS, facilitate the recognition and characterization of gene fusions, with the generation of full-length transcripts allowing for the identification of the genomic regions involved. This overcomes the challenges faced by short-read technologies, where chimeric reads or discordant read pairs make it difficult to identify the products of gene fusion (87). While there are a lack of studies showing the utility of LRS for gene fusions in CNS cancer, other studies have applied LRS techniques successfully to detect gene fusions in cancer research (87–89).

### 5.2 Transcriptomics

Another valuable application of LRS in cancer research is in the context of alternative splicing, a genetic process involving the creation of different mRNA isoforms by selecting different splicing sites from the same gene (90). This process plays a fundamental role in generating proteomic diversity, with the proteins generated potentially dictating the biological behavior of a cell, such as cellular growth. Importantly, splicing patterns can change the reading frame of mRNA, resulting in the encoding of different isoforms of proteins, or in the downregulation of critical untranslated regions with relevant regulatory sequences (91). The alteration of this genetic mechanism is pivotal in oncogenesis, especially in brain cancer, where the brain is one of the organs with the highest rates of alternative splicing due to its contributions to the nervous system development (92, 93). For example, a study by Kim et al. demonstrated how a nuclear speckle protein, responsible of facilitating RNA splicing, had the highest rate of aberrant upregulation in GBM, with a correlation between the abnormal upregulation of this protein and patient survival (93). LRS can help identify abnormal alternative splicing by sequencing mRNA or complementary DNA, as it allows for the identification of different isoforms of genetic material (80).

Likewise, the use of long-read technologies can be beneficial for the detection of non-coding RNAs (ncRNAs), such as long non-coding RNAs (lncRNAs), microRNAs or circular RNAs (80). Over 98% of the human genome is transcribed into ncRNA which plays various roles in cellular functions, such as post-transcriptional gene regulation (94, 95). Despite being initially considered “transcriptional noise”, technological advances have revealed the involvement of ncRNAs, such as lncRNAs, in cancer pathogenesis (96). For example, the product of the H19 gene, a lncRNA located on chromosome 11p15.5, is highly expressed in high-grade gliomas, modulating angiogenesis, cellular growth, proliferation, invasion, drug resistance and radiation resistance (97–102). In terms of LRS, Nanopore direct RNA sequencing can be effectively used to identify ncRNAs without the need for cDNA conversion or amplification, although other techniques are also commonly used (103).

One of the advantages of LRS lies in its ability to investigate nucleic acid modifications. Short-read technologies in epigenetics are limited, struggling to accurately map repeated sequences, and facing constraints in haplotyping. LRS technologies, however, offer improved results in identifying DNA modifications, allowing for the detection of various nucleic acid modifications such as DNA 5methylcytosine (5mC), RNA N6-methyladenosine (104, 105), or 8-oxo-7,8-dihydroguanine (OG) (106) within a single read. In a study done by An. et al., an α-hemolysin (α-HL) nanopore sequencing demonstrated the versatility of this technology by accurately detecting OG, a biomarker of oxidative stress, within G-quadruplex structures from the human telomere sequence (106). The impact of DNA and RNA modifications on gene expression has been demonstrated in different diseases, including cancer and neurological syndromes (107). For example, RNA methylation has gained importance over the recent years due to its association with cancer biology (108). Particularly important, m6A methylation has been linked with cancer progression, as this modification directly influences several steps of RNA metabolism (e.g., RNA expression), leading to the regulation of different cellular processes. When aberrant, these processes contribute to tumorigenesis, affecting apoptosis regulation, cell proliferation, cell invasion and cancer metabolism (109, 110). In CNS tumors, specifically GBM, m6A methylation has shown to have a key role in tumorigenesis and self-renewal of malignant cells (111). While the applications of epigenetics and LRS are discussed in a subsequent section, it is essential to note how epigenetic alterations directly contribute to oncogenesis. Changes in gene regulation significantly affect carcinogenesis processes including cell growth, proliferation and immune evasion (112–115). For instance, epigenetic alterations of DNA repair genes, such as O6-methylguanine-DNA methyltransferase (MGMT), which plays a significant role in CNS tumors like GBM, can predispose mutations in key genes such as p53 (116). Similarly, genetic mutations in epigenetic modifiers are hypothesized to induce abnormal epigenetic changes like abnormal DNA methylation, histone modifications, and alterations in nucleosome positioning (116). The interdependence of genetic and epigenetic alterations gains more relevance as our understanding of cancer improves. The simultaneous analysis of genetic and epigenetic mutations proves invaluable for understanding tumor carcinogenesis, as these two factors interact (117, 118). The abnormal interaction between the genotype and epigenotype of a cell inevitably results in a variety of human diseases, including cancer (118).

### 5.3 Single-cell sequencing

With the revolution of single-cell sequencing (SCS) technologies, highly heterogeneous populations within a tissue (e.g., tumor biopsy) can be extensively analyzed with a high-resolution using these techniques. Epigenetic information within a particular population can be inferred using SCS, which could provide information regarding the DNA methylome (119, 120), transcriptome (121–124), histone modifications (125–128), among others. Traditionally, SCS has been conducted using NGS platforms. However, with the advent of LRS, SCS analyses are possible using LRS platforms. For example, in a study conducted by Chang et al. a multi-omics analysis was conducted on genome and transcriptome sequencing information using a LRS platform (129). The study used this technology for the analysis of genomic structural variations within single cells and found to be highly reliable, as extrachromosomal DNA was mapped in heterogeneous cell populations and in clinical tumor samples.

## 6 Current application of LRS in neuro-oncology

**Figure 1:**
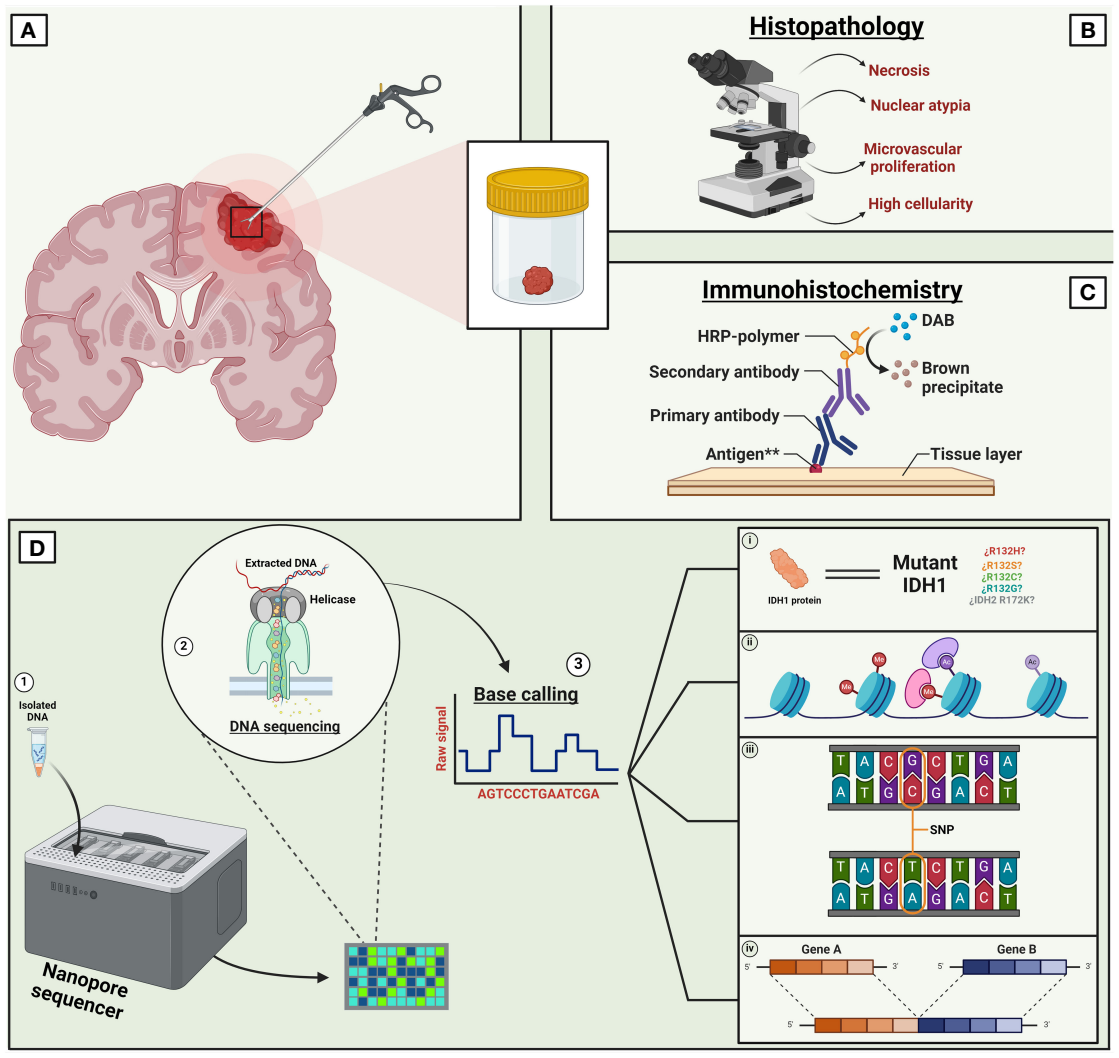
Current paradigm of long read sequencing in CNS tumors. Traditionally, the diagnosis of CNS tumors relied on the histopathological analysis of a tissue biopsy **(A)**. However, the contemporary and standard diagnosis of CNS tumors requires an integrated approach. Combining a tissue-based histological examination **(B)** with molecular diagnosis involving immunoreactivity tests **(C)** and advanced new generation technologies like LRS **(D)**. LRS has the capability of screening a wide variety of molecular changes such as multiple mutations, mutants’ variations (i), methylation modifications (ii), single nucleotide variants (iii), gene variants (iv), among others. Other LRS technologies such as ‘single molecule real-time’ are not shown in this figure. **Immunoreactivity for antigens such as cytokeratin, neurofilament protein, glial fibrillary acidic protein, etc. IDH, Isocitrate dehydrogenase, DAB, Diaminobenzidine, SNP, Single nucleotide polymorphism. Created with Biorender.com.

The current approach to diagnosing CNS tumors relies on an integrated diagnosis provided by the histopathological and molecular classification of a sample to aid in the decision-making and in the establishment of personalized treatment plans (**Figure 1**). The fast progression of LRS technologies has yielded promising results, as demonstrated by several studies that have highlighted the utility of this technique in clinical neuro-oncology practice (**Supplementary Table 1**).

For instance, Wongsurawat et al. showed the feasibility of utilizing nanopore Cas9-targeted sequencing (nCATS) (130) in four human cell lines and in eight fresh brain tissue samples from patients diagnosed with gliomas. They successfully assessed the status of two molecular biomarkers (MGMT methylation and IDH1/2 mutations) within 36 hours (27). In this study, the use of nCATS enabled simultaneous evaluation of both genetic mutations (IDH status) and epigenetic modifications (MGMT methylation). The results were comparable to traditional diagnostic methods like Sanger and Illumina sequencing for IDH status, as well as pyrosequencing and methylation-specific PCR for MGMT methylation. Furthermore, the study demonstrated the utility of nCATS in identifying single nucleotide variants (SNVs) in MGMT and IDH1/2 loci. All accomplished within two days of specimen collection and at considerably lower cost than traditional methods.

Similarly, in another study conducted by Wongsurawat et al. a “nanopore-based copy-number variation sequencing “ (nCNV-seq) was used to evaluate three different *in vitro* glioma cell lines (BT88, HOG and U87 cells) and 19 IDH-mutant patient derived gliomas (131). In this study, nCNV was employed for assessing the status of the cyclin-dependent kinase inhibitor 2A/B (CDKN2A/B) along with the codeletion of 1p/19q. In the cell lines, nCNV-seq not only showed the same genetic profile as the nanopore-based whole genome sequencing (WGS), but also provided faster and more accurate results in as little as 8 minutes (compared to 250 min with WGS). Furthermore, nCNV-seq was compared with an EPIC array, Illumina WGS, and FISH test for analyzing DNA methylation, copy number variations (CNVs) and chromosomal deletions, respectively. nCNV-seq demonstrated the same results as the other methods with a high concordance rate (EPIC array 11/11, Illumina WGS 8/8 and FISH 7/7) (131). The study concluded how this LRS platform showed promising results in rapidly detecting relevant genetic alterations in a CNS tumor, being concordant with other diagnostic and more commonly used methods. Interestingly, other studies have also shown high concordance rates when comparing this technology to traditional methods. For example, Djirackor et al. conducted a study assessing six independent cohorts comprising 105 tissue biopsies from patients with CNS tumors, using nanopore whole-genome sequencing for DNA methylation analysis (NDMA) and compared the results with the methylation-based classification of the integrated diagnosis with neuropathology. Importantly, this approach showed concordance with final pathological diagnosis in 89% of the cases, showing high intraoperatively accuracy with better results than standard frozen section analysis. Furthermore, this study was the first to demonstrate feasibility of obtaining intraoperative diagnosis as the results could be accomplished prior to the end of neurosurgical resection allowing for modification of surgical plans as needed (132).

Despite the limitations of these technologies, such as a high error rate with certain genetic alterations like SNVs or short insertions and deletions (InDels) (133), various applications of LRS can be implemented in clinical practice to further improve cancer diagnosis. Importantly, combining emerging technologies, such as artificial intelligence, with sequencing data can further expand the amount of information that can be obtained in a more cost-and time-effective manner (134). In neuropathology, different studies have shown how this technology could be beneficial when used intraoperatively to characterize and classify CNS tumors rapidly. For example, a recent study done by Vermeulen et al. incorporated artificial intelligence by developing a “patient-agnostic transfer-learned neural network” trained on simulated and real nanopore sequencing data, with over 40 million of sequencing runs. By using nanopore-sequencing data, this neural network (“Sturgeon”) was capable of discerning the subclassification of CNS tumors, in real time, within 1.5 hours from tissue collection in both adult and pediatric patients; accurately classifying 72% of the samples (135). Additionally, this timeframe showed to be compatible within the operative time, demonstrating the high utility and applicability of LRS with modern technologies within a short timeframe to produce accurate results. The use of a machine-learning diagnosis-based model in an intraoperative setting demonstrated how the implementation of AI with LRS data in a timely manner, can potentially aid with surgical decision-making and thus potentially improving patients’ outcomes. Similarly, in a study done by Kuschel et al., a random forest classifier pipeline (“nanoDx”) was used for DNA methylation-based classification of 382 brain tumor biopsies using nanopore-lowpass whole genome sequencing data. In this study, nanopore-based methylation was concordant with 81.4% of the samples when comparing with methylation array-based classification, demonstrating a reliable classification for CNS tumors (136). On the other hand, while LRS is not the gold standard technique for the sequencing of tumor samples in neuropathology, the combination of this technology with other sequencing techniques, such as NGS, could provide a more proficient characterization of tumors by exploiting the strengths and covering the weakness of each technology. In 2023, Zwaig et al. used linked-read sequencing (LRS with short read sequencing) for a comprehensive analysis of medulloblastoma genomes (137). With the use of long-range information from LRS together with the high base pair accuracy of short-read sequencing, the authors were able to characterize different genomic variants such as SVs, CNVs along with the first known detection of extrachromosomal DNA using this methodology.

Neurosurgical studies have also shown how LRS technologies could be integrated into clinical practice by showing a rapid molecular diagnosis and outperforming traditional methods such as “frozen section analysis” in selected and challenging clinical samples (132). This aspect is highly relevant in a surgical setting given that in most cases the extent of tumor resection is variable and strictly correlates with the classification of the tumor. The optimal surgical strategy for approaching brain tumors relies on accurate molecular diagnoses, and it is not the same across tumor types or subgroups (132). Prognostic factors of different tumors depend on the extent of resection, such is the case for atypical teratoid/rhabdoid tumors (138). Moreover, differentiation between tumor subgroups could have a critical impact in prognosis and survival. For example, mesenchymal recurrent IDH-wildtype GBM has not shown a survival benefit with gross total resection (GTR) in comparison with the receptor tyrosine kinase (RTK I and II) subclass, which indeed benefits from GTR (139). Similarly, in medulloblastomas, GTR has a different impact in survival rates between subgroups. GTR has no impact on the survival rate of WNT-activated medulloblastomas, whereas group 3 medulloblastomas show a survival benefit from GTR (140). Other tumors such as papillary craniopharyngiomas with a targetable BRAF V600e mutation can determine the surgical strategy of a patient (141). Importantly, intraoperative subclassification of brain tumors with novel profiling methods, such as methylation-based classifications, can facilitate and further expand these applications. For example, both retrospective and prospective studies have shown that the stratification of meningiomas with methylation-based classification can be considered a strong prognostic predictor across subtypes and can singularly outperform the current WHO grading system (142–148). When removing meningiomas around critical structures such as in the skull base, the knowledge of recurrence likelihood and/or response to radiation may influence the extent of resection. Similarly, in the study done by Djirackor et al., intraoperative NDMA classification showed how the surgical strategy would have been modified in 12 out of 20 patients. For example, in one patient, surgery was halted due to inconclusive imaging and frozen section results suggestive of lymphoma. However, the patient had to be reoperated as final pathological analysis showed the presence of a SHH subtype medulloblastoma; this diagnosis was concordant with the initial intraoperative NDMA (132). Importantly, NDMA results were obtained within 120 minutes of tumor biopsy, discerning CNS tumors and providing guidance in difficult imaging and/or frozen section analysis specimens (132). Therefore, rapid intraoperative diagnosis can positively impact the outcome of patients giving valuable molecular information to the surgeon. These applications will likely continue to expand with the improvement of the available tools for genomic-based brain tumor prognostic stratification. Similarly, LRS technologies have shown to be rapid, accurate and proficient when incorporated in an intraoperative workflow (132) with the possibility of sequencing for intraoperative formalin-fixed, paraffin-embedded tissues (FFPE). Mimosa et al. validated a nanopore-based IDH mutation assay for glioma samples in FFPE tissue (149). In this study, nanopore sequencing was used on 66 glioma cases in which IDH mutational status was known, demonstrating an accuracy of 100% for SNVs detection when comparing with traditional methods. The assay showed an analytical specificity and sensitivity of 100% within a short period of time, with low sequencing costs and with minimal infrastructure required. Moreover, the rising importance of DNA methylation profiling in oncological practice, which will be further dissected in the following section, has led to the integration of methylation arrays with traditional tissue preservation methods such as FFPE and fresh-frozen samples (150, 151). Although novel methylation arrays have shown promising results in the diagnostic workflow of brain tumors, especially in rare tumors that have not been yet defined by characteristic mutations such as in astroblastomas or spinal cord gliomas (152–154), these technologies have higher turnaround times and impose a higher cost than some of the traditional techniques used during the routine CNS tumors clinical workflow (8, 150). However, the implementation of nanopore sequencing for methylation profiling and copy number variation analysis has shown to be feasible for implementing a rapid methylation-based CNS tumor classification in both cryopreserved and FFPE tissues (132, 135, 136, 150). Nonetheless, further studies are needed to determine the reproducibility of this technique as methylation profiling with LRS has only been applied to samples with high-quality DNA (150). It is clear that the versatility of this technology can be used in further and more complex genomic alterations, which are known to be characteristic of CNS tumors. This is particularly relevant to epigenetics modifications, a mainstream topic in recent years, as advancements in detection technologies have led to the discovery and understanding of the different factors that drive normal cells to become cancerous. In CNS tumors, these alterations hold a special interest.

### 6.1 Importance of epigenetics in neuro-oncology

As previously mentioned, epigenetics plays a crucial role in carcinogenesis (116, 155). This term, which was initially introduced by Conrad Waddington in 1942 as “the branch of biology which studies the causal interactions between genes and their products, which bring the phenotype into being” (156, 157), has been extensively studied in the recent years as there are known mechanisms that modify chromatin structure and have been reported to be crucial in various and aggressive CNS tumors such as GBM (158–160), medulloblastoma (161–163), ependymoma (164, 165), diffuse intrinsic pontine gliomas (166, 167), meningioma (147), among others. The epigenetic modifications that are capable of modifying chromatin structure are encompassed into four categories: DNA methylation, histone modifications, and non-covalent mechanisms (e.g., nucleosome remodeling, non-coding RNAs) (155). All these modifications complement each other and are known be part of what is called the “epigenome”. Normally, the regulation of these mechanisms works in normal cells as a mean of genome regulation by “restricting” or “facilitating” chromatin accessibility, and thus regulating gene expression. However, these mechanisms get mutated and distorted in abnormal cells, contributing to the initiation and progression of cancerous cells (118). One of the most relevant epigenetic modifications in CNS tumors is the DNA methylation of cancerous cells; many tumors possess a unique methylation profile reflecting the complex genetical alterations from the cell of origin, giving the cell a unique “barcode” (168). The cancer methylome, which is characterized by “genome-wide hypomethylation and site-specific CpG island promoter hypermethylation” (155) and which represents the blueprint of the “somatically acquired DNA methylation changes” of precursor cells (13), is an important biomarker that can be used for stratifying tumors into subgroups and better predicting treatment responses (169–171).

With the new modifications of the WHO guidelines (18), the neurosurgical paradigm of CNS tumors shifted and started relying on the molecular profiling of DNA methylation profiling of tumors. The study of the cancer methylome, is an unquestionably potent tool for the stratification of CNS tumors. It is considered a reliable method for the classification of several CNS tumors (13), such as pediatric brain tumors (169), diffuse gliomas (172) and other diagnostically challenging cases (173). Importantly, characterizing CNS tumors, such as in the pediatric population, has shown its reliability for obtaining a better prognostication and a more accurate response to treatment (171, 174), being easily reproducible using fresh-frozen or formalin-fixed paraffin-embedded (FFPE) tumor samples (175). For instance, in a study by Afflerbach et al., 40 FFPE samples derived from CNS tumors, with an average storage duration of 19 months, were classified based on a methylation analysis by implementing two publicly available methylation pipelines (nanoDx and Sturgeon) (135, 136) on the nanopore sequencing data of these samples (150). In this study, nanoDx and Sturgeon classified 50% and 85% of the samples into the correct methylation class, respectively. Additionally, out of the 40 FFPE samples, 16 had poor-quality DNA and had higher storage times. Interestingly, Sturgeon classified 88% of these samples correctly, demonstrating that nanopore-based methylation classification is feasible with low-quality DNA samples. The turnaround time in this study also showed promising results when compared with methylation arrays showing turnaround times of 3-4 days by using the Illumina EPIC array *vs <*6* h* after DNA extraction with the proposed protocol in this study (150).

Utilizing a DNA-methylation based classification of tumors could go beyond the clinicopathological classification by providing a deeper understanding. For example, methylome data could be sufficient for the correct classification of tumors such as meningiomas (143). Nevertheless, the use of traditional diagnostic methods such as conventional histopathology together with a complete molecular profile (including DNA methylation) could improve the approach to CNS tumors classification as it has shown to have a positive impact by modifying the definitive diagnosis in some patients (8, 176, 177).

The implementation of other ongoing and innovative technologies such as LRS for DNA methylation profiles not only complements the traditional classification methods but gives a more refined and standardized CNS tumors classification for physicians and researchers, improving patient management. The impact of this classification has been evident in population-based studies. Pickles et. al, assessed the impact of implementing a DNA methylation-based classification into diagnostic practice of two large pediatric cohorts. Concordantly, methylation profiling of CNS tumors in this study modified the initial diagnosis by subclassifying 35% of the tumors in the studied population with an estimated effect on the traced management in 4% of the patients (176).

LRS technologies, particularly nanopore sequencing, prove to be valuable in the identification of base modifications due to the high sensitivity of these devices to the electronic currents generated by base modifications (104, 178). In neuropathology, different studies have effectively evaluated the methylome of CNS tumors using LRS. In 2017, Euskirchen et al. utilized a MinION platform for a “low pass” whole genome sequencing to generate and evaluate copy number, SVs, and methylation profiles of CNS tumors (179). By comparing the methylation events identified by the nanopore platform with the matched methylome microarrays, they were able to detect a correlation between the single-read methylation status of given CpG sites with the equivalent beta value in the microarray data. Furthermore, the authors used an *ad hoc* random forest classification of 7 glioma samples using CN alone, methylation only and both profiles together; finding an improved overall precision of sample classification by the combination of both approaches. In this study, methylation data was sufficient for the subclassification of gliomas, and demonstrated the feasibility for distinguishing the origin of a tumor within a few hours, which has been shown to improve the diagnoses of cancers of unknown primary (e.g., primary brain tumor *vs* brain metastases) (180).

The use of LRS such as nanopore sequencing for methylation-based studies, has shown to be highly feasible. This technology provides an accessible way to assess the methylome of a tumor due to its intricate sensitivity to base modifications such as 5-methylcistosine (5-mC), 5-hydroxymethylcytosine (5-hmC), N^6^-methyladenosine (m6A) and N^5^-methylcytosine, distinguished by alterations in the current signal in these sequencing devices (104, 105, 181–184). The potential of LRS makes this technology feasible for expanding coverage by identifying additional base modifications. Therefore, it stands as a great option for the application of these technologies in providing the complete DNA methylation profile of CNS tumors.

### 6.2 Liquid biopsies and LRS in neuro-oncology

While direct biopsies serve as the primary method for identifying the histological and molecular features of a tumor, there are several limiting factors that need to be considered, especially when attempting to comprehensively characterize an aggressive tumor. Despite various improvements in surgical techniques and imaging technologies, the heterogeneity of this pathology makes it challenging to obtain a high-quality sample that could accurately represent its complete and precise genomic profile. This challenge is particularly evident in highly aggressive primary and metastatic tumors, as they are known to exhibit genomic diversity from clonal heterogeneity as well as high mutational burden (185). Even with the most thorough gross total resection, it will only represent a specific moment in time, which is why treatment plans are dynamic as the genomic phenotype of an aggressive tumor will not always be the same, highlighting the inherent limitations of static treatment plans (186, 187). Moreover, downsides of invasive procedures, such as surgical complications (e.g., bleeding, infection, need for reintervention, post-operative neurological deficit, etc.), intrinsic comorbidities or patient risks further complicate the acquisition of an exact, precise, and safe tumor tissue sample. Therefore, research efforts have been invested in less invasive procedures that could accurately give a solution for a safe and effective diagnosis, characterization, follow-up, and treatment response of a tumor. One of the recent methods that has been having more relevance are liquid biopsies.

Liquid biopsy, refers to the collection of body fluids (such as cerebrospinal fluid or venous blood) for the identification of “tumor-derived nucleic acids” (188). These nucleic acids are known to be shed by brain tumors into peripheral fluids and have been previously identified with ‘peripheral’ sampling (189–191). The discovery of circulating cell-free DNA in cancer patients opened a way to a non-invasive method for the genomic profiling of tumors, avoiding interventional biopsies and expanding the possibilities of multiple and serial evaluations throughout the evolution of the disease (192). Different studies have shown how particular mutations can be detected in different body fluids, such as serum or in plasma (193–195). It is worth noting that DNA may be shed into the bloodstream at lower rates compared to other types of tumors due to the presence of the blood-brain barrier. This may result in a limited quantity of circulating DNA, making the identification of DNA mutations, especially those occurring at low frequencies, quite challenging. Importantly, the genetic material of a tumor can be found as circulating tumor cells, extracellular vesicles and cell-free nucleic acids (188).

NGS has been used in several clinical studies as it has been proven to be successful in isolating tumor derived nucleic acids in body fluids such as CSF (191, 196). Nevertheless, LRS technologies have also shown to be successful in detecting molecular alterations in liquid biopsies (**Supplementary Table 1**). The preference for NGS instead of LRS is attributed to the higher error rate of the latter and the low number of studies that have used this technology for liquid biopsies. Even so, LRS has potential advantages in liquid biopsies relative to NGS. In one study done by Bruzek et. al, ultra short CSF cf-tDNA fragments were analyzed with LRS in 12 pediatric patients with diagnosis of high-grade gliomas (pHGG). Nanopore sequencing of CSF showed a sensitivity and specificity of 85% and 100%, respectively, with a remarkably low amount of DNA needed in comparison with NGS (15 nanograms *vs* 30-45 nanograms of input DNA), and successfully detected the H3F3A K27M mutation with only 0.1 femtomoles needed of cf-tDNA (197); with this results showing how nanopore sequencing is an efficient and sensitive approach that is similar to NGS for liquid biopsies. Additionally, this LRS technology showed to be highly efficient for the sequencing of cf-tDNA as it took approximately 12 hours to get results from the time of the lumbar puncture to the identification of the variant allele fractions of the SNPs. Finally, the authors demonstrated the utility of LRS in serial monitoring for patient specific mutations. In this study, 2 patients were enrolled in a clinical trial for a new drug against pHGG. In both cases CSF was sequenced on 3 points of time, accurately reflecting the molecular response over time with the new drug (197).

Similarly, a recent study done by Afflerbach et al. used low-coverage nanopore sequencing for CNVs and methylation profiles of 129 CSF-derived cfDNA samples, which were collected in different points of time (pre-surgery, early-post surgery and later after surgery) (198). The cohort collected by the authors consisted of 22 different entities, with medulloblastoma being the most predominant CNS tumor and with the population consisting of children or adolescents. In pre and early post-surgery CSF samples, nanopore sequencing was able to detect cfDNA in 45% of the samples. Interestingly, post-surgery CSF samples demonstrated how in 2 patients, the detection of cfDNA using nanopore sequencing, orientated towards disease remission or relapse based on the new genetic alterations seen in the CSF methylation profiles. Remarkably, this study confirmed the usefulness of liquid biopsies for minimal residual disease detection and the validity of nanopore sequencing for detecting cfDNA in CSF samples of several CNS tumors, displaying the potential of this technology for sequencing cfDNA from CSF samples for a complete approach of CNS tumors.

These studies show the utility of the implementation of LRS for liquid biopsies. The possibility of acquiring accurate real-time results of liquid biopsies during different periods of time in a single patient, could better predict and assess a patients’ molecular status throughout an established treatment or during follow-up. Furthermore, the low input genomic material needed for samples could be particularly useful in selected patients where there is a difficulty of obtaining large volumes safely, such as pediatric patients. Additionally, liquid biopsies have shown promising results that are yet needed to be examined on future clinical trials that could showcase the utility of this technology for its implementation into routine diagnostics of CNS tumors.

## 7 Current research gaps

Although LRS has shown promising results in the genetic study of cancer, these technologies still have limitations. One of the main drawbacks consists of the high error rate that these technologies show, thus limiting the accuracy of the data produced (80). Importantly, the high error rate observed in these devices can be attributed to the low sequencing depth of LRS, with reported sequencing error rates of 10-15% in SMRT and 5-20% in nanopore sequencing (199). One of the solutions for this drawback is the implementation of short-reading sequencing with long-reading sequencing, as it can importantly improve data analysis by having better accuracy, such as in the study did by Zwaig et al. (137). As most genetic studies on cancer have focused on other more used and well-known technologies such as NGS, there is a necessity for the continuous improvement of LRS in terms of tools for data analysis such as the elaboration of new algorithms for better analysis of longer and complex reads. Importantly, there is a need for validation in more and larger clinical trials that could standardize the use of these technologies into the daily clinical practice. Although LRS have been constantly improving there is still skepticism given by the high error rate displayed by this technology when it was first introduced (200, 201). Recent base-calling algorithms of these technologies for DNA sequencing are highly accurate, with SMRT having an accuracy of 99.9% and NS of 99.6% (202, 203), which is a noticeable contrast with the previous ~85% of NS when this platform was first presented (202), and also with the accuracy rates of NGS devices such as Illumina (>99.9%) (41). However, despite the increasing accuracy of LRS over the years, error correction is still a major challenge for LRS analysis (204). Unfortunately, sequencing technologies are still expensive, representing disparities in the diagnosis of CNS tumors in developing countries where approximately 70% of cancer deaths occur in these countries (205), possibly being attributed to the low and outdated cancer infrastructure, leading to delayed and incomplete or inaccurate diagnoses (206). The importance of globalizing medicine, implies accessibility all over the world, given the possibility to low- and middle-income countries to afford these new technologies so that the most updated and latest guidelines could be applied in terms of cancer diagnosis, treatment, and prognosis. Therefore, devices such as LRS technologies, which require minimal infrastructure could be useful in addressing global cancer disparities.

A relevant limitation of this technique is related to specimen selection. Intraoperatively surgeons will need to collaborate with pathologists to ensure that representative tumor samples that are diagnostic are sent for analyses. Intraoperative LRS should be intended in selected cases where rapid subgrouping of an entity could determine an approach that drastically improves the patient’s prognosis, such as determining the extent of resection. In certain classifications, such as the methylation-based classification of IDH-wildtype GBM, the use of LRS is particularly important in intraoperative settings due to its benefits for surgical management (139). Papillary craniopharyngiomas can have the actionable mutation BRAF V600e and respond well to targeted therapy. Therefore, the intraoperative knowledge of knowing that this targetable mutation is present may change the surgical approach and decrease the risk of injury to critical neurovascular structures as these tumors are often closely associated with the optic nerve complex (141). However, in tumors where subclassification does not significantly influence management strategies, the use of LRS technologies is not beneficial. Therefore, it is crucial to appropriately determine the correct use of LRS in select cases where patients will benefit.

## 8 Future directions

**Figure 2:**
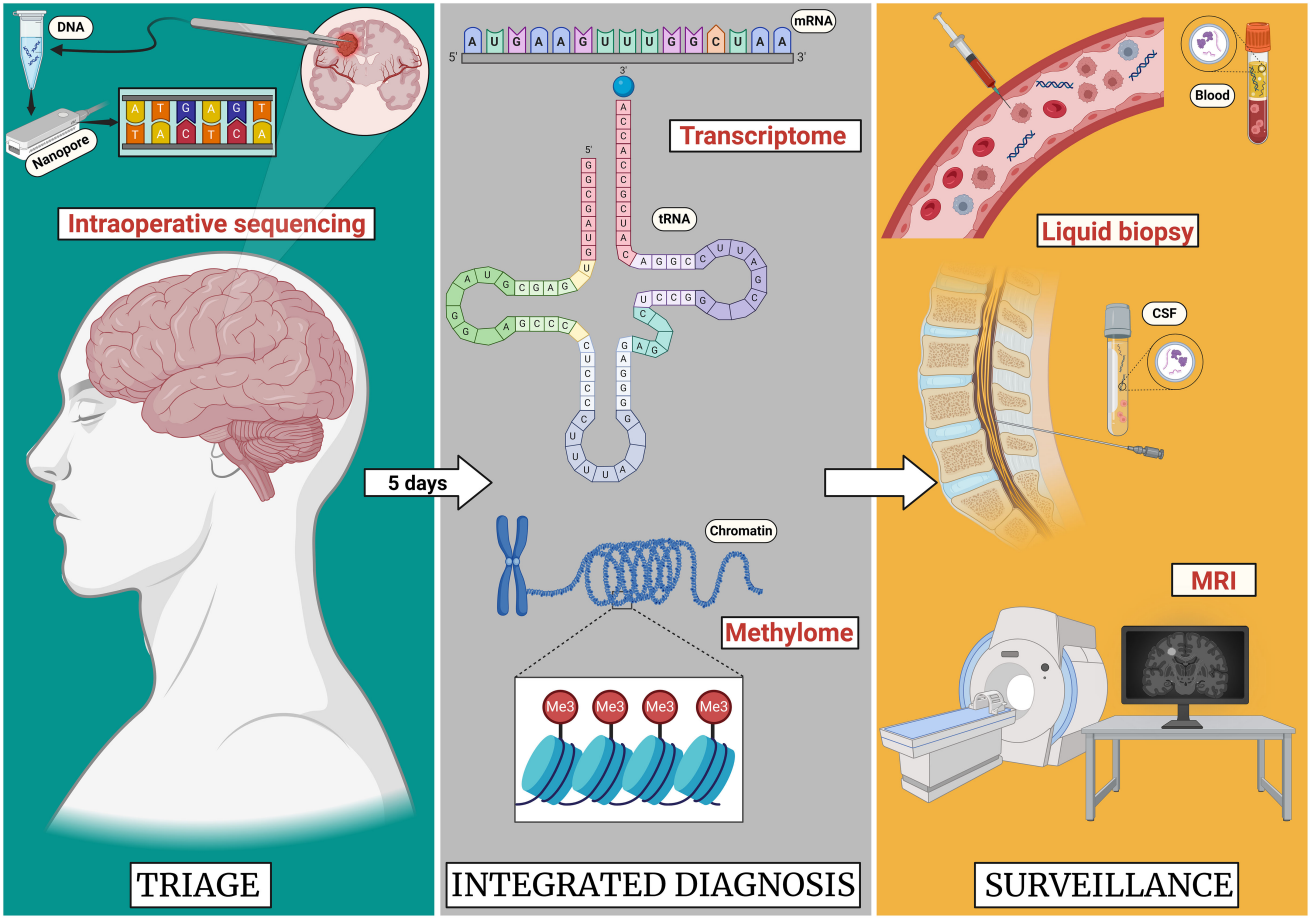
Proposed future paradigm of long read sequencing in the application of CNS tumors. As the sequencing technologies improve, the advances in the neurooncological practice will evolve. In the case of a brain tumor resection, these sequencing technologies could allow for the intraoperative diagnosis of the tumor allowing for decision making on extent of resection during the surgery. While in 5 days a complete transcriptome and methylome profile of the tumor could be obtained providing improved prognostication. Lastly follow-up surveillance will likely include LRS for liquid biopsy in combination with standard MRI surveillance. Created with Biorender.com.

As technology advances and diverse studies show the clinical utility of these new technologies, the integration of these tools in clinical practice could be differential when treating CNS tumors. The possibility of real-time intraoperative diagnosis could not only give a more accurate characteristic of the genomic profile of a particular tumor, but it could be a better guide for intra-operative surgical decision making for determining how aggressive to be with resection (132). However, it is relevant to view the inclusion of this technology in the field of neuro-oncology as a part of an integrated multi-technique diagnostic arsenal rather than an exclusive and one-way technique for categorizing brain tumors. With the use of rapid LRS technologies, approaching a CNS tumor in a global manner, with adjunct tools such as preoperative imaging, intraoperative frozen sections, and other genomic techniques, will permit a better understanding and more accurate characterization of the complicated biological background across entities. This will open more opportunities for managing this disease in the most beneficial way possible for patients. Faster genomic results will also allow for more rapid patient stratification for precision medicine trials. These could modify the current neurosurgical approaches of CNS tumors. For example, the use of relevant genetic information such as the tumor methylome, could arguably be one of the best and most accurate ways of characterizing and giving prognosis for a tumor. Furthermore, the implementation of a DNA methylation-based classification of CNS tumors has shown to improve the diagnostic precision in cancer samples, demonstrating an impact in the molecular subgrouping and final treatment of cancer patients. With this information, patients would benefit from knowing early personalized surgical and or adjuvant treatments. Additionally, the use of these new sequencing technologies could also impact on patients follow up, as one of the promising applications of these techniques is with liquid biopsies. The improvement of detection techniques and understanding of tumor derived nucleic acids, could modify tumor surveillance methods. Oftentimes, it is difficult to determine treatment related changes from recurrence using radiographic imaging alone. Determining the molecular profile of a patient within the evolution of the disease, with a less-invasive procedure, could lead to more quantitative follow-ups given the knowledge of the exact genotypic information of the tumor in terms of new molecular alterations, treatment response or recurrence. The correlation of this data with imaging such as MRI, could more accurately determine the need for additional interventions, thus giving personalized treatment to each patient with the hope of improving their prognosis and future outcomes (**Figure 2**).

## 9 Conclusions

The emergence of long read sequencing technologies has shown promising results in the integrated management of CNS tumors. The ultrarapid sequencing-based diagnosis of this technology allows for a timely molecular classification of a tumor within minutes of tissue sample availability; expanding the possibilities and strategies that could be used to approach the tumor intraoperatively. Although there are still several challenges with the use of LRS, such as the ones already mentioned, the combination of this technology with previous ‘more established’ ones such as NGS, could drastically modify the outcomes of a patient by providing a more accurate and rapid diagnosis. This improvement with diagnosis can lead to better patient outcomes and provide further opportunities for precision medicine. With the advancements in the therapies available for CNS tumors, the use of this technologies will be fundamental, as a complete and exhaustive molecular understanding and diagnosis of a complex tumor will allow for personally tailored treatment. The cost-effectiveness of these and further developed sequencing technologies will be of great use in developing countries, improving the worldwide diagnosis, and needed treatment for cancer. Unfortunately, there is still a big gap between developed countries and developing countries in terms of diagnosis and ultimately, treatment. The imperative need for significant global investments in cancer treatment, developing affordable and effective diagnostic and treatment tools, is evident. Genomics can be used as a tool to address cancer disparities (207). The integration of LRS into clinical neurooncology paradigms is on the horizon and can lead to significant enhancements in diagnosis, prognosis, therapeutic management, and health equity.

## Author contributions

WS: Investigation, Visualization, Writing – original draft, Writing – review & editing. SZ: Investigation, Writing – original draft. JN: Validation, Writing – review & editing, Investigation. MG: Investigation, Validation, Writing – review & editing. MB: Investigation, Validation, Writing – review & editing. KR: Investigation, Validation, Writing – review & editing. CW: Investigation, Validation, Writing – review & editing. OV: Investigation, Validation, Writing – review & editing. AR: Conceptualization, Investigation, Resources, Supervision, Validation, Writing – original draft, Writing – review & editing.
