# Supplementary material for: Long-read sequencing for brain tumors
Source: Front Oncol. 2024 Jun 10;14:1395985. doi: 10.3389/fonc.2024.1395985 (PMC11194609; doi:10.3389/fonc.2024.1395985)
Supplement: Supplementary file 1 [file Table_1.docx]

**Supplementary table 1. Published articles using third-generation sequencing technologies in CNS tumors.**

| **Study** | **Year** | **TGS method** | **Procedure** | **Type of CNS tumor** | **Compared with other technologies** | **Remarks** |  |  |
| --- | --- | --- | --- | --- | --- | --- | --- | --- |
| Afflerback et al. | 2024 | Whole genome nanopore sequencing | Tumor biopsy | Glioblastoma, oligodendroglioma, posterior fossa ependymoma, medulloblastoma, pilocytic astrocytoma and meningioma | Yes | Demonstrated the feasibility of LRS in FFPE samples for methylation-based classification of CNS tumors and generation of genome-wide copy-number profiles |  |  |
| Vermeulen et al. | 2023 | ONT MinION + Neural network classifier ("Sturgeon") | Tumor biopsy | Pediatric tumors  Adult tumors (diffuse gliomas) | No | Created a neural network based on TGS capable of discerning tumor types within 1.5 hours of tissue collection |  |  |
|  |  |  |  |  |  |  |  |  |
| Zwaig et al. | 2023 | Long-read Nanopore and PacBio sequencing | Tumor biopsy | Medulloblastoma | Yes | Combined long-read sequencing with short-read sequencing, detecting complex SVs, PMs, extrachromosomal DNA, and new complex genetic events |  |  |
|  |  |  |  |  |  |  |  |  |
| Afflerbach et al. | 2023 | Nanopore (MinION) sequencing on cell-free DNA | Liquid biopsy (CSF) | 22 brain tumor (sub)types (WHO 2021) | Yes | TGS was able to detect ctDNA in different tumor subtypes and disease stages |  |  |
|  |  |  |  |  |  |  |  |  |
| Rausch et al. | 2023 | ONT long-read sequencing | Tumor biopsy | Medulloblastoma | Yes | Discovered a new complex SV pattern and an allele-specific methylation using long-read sequencing |  |  |
|  |  |  |  |  |  |  |  |  |
| Mimosa et al. | 2023 | Nanopore-based IDH mutation detection assay using glioma formalin-fixed, paraffin-embedded tissue (FFPE) | Tumor biopsy | Diffuse glioma | Yes | First study validating a diagnostic assay for IDH SNVs detection using a nanopore-based method in FFPE tissue |  |  |
|  |  |  |  |  |  |  |  |  |
|  |  |  |  |  |  |  |  |  |
| **Study** | **Year** | **TGS method** | **Procedure** | **Type of CNS tumor** | **Compared with other technologies** | **Remarks** |  |  |
| Wongsurawat et al. | 2023 | Nanopore-based copy-number variation sequencing (nCNV-seq) approach with SMURF-seq protocol modification | Tumor biopsy | IDH-mutant gliomas | Yes | nCNV-seq rapidly and accurately identified CDKN2A/B homozygous deletions with 100% accuracy and 1p19q co-deletions with an accuracy of 89.4%, showing strong concordance when compared to other technologies |  |  |
| Kuschel et al. | 2023 | Nanopore low-pass whole genome sequencing | Tumor biopsy | 40 brain tumor (sub)types (WHO 2016) | Yes | This TGS technology accurately classified brain tumors based on their methylome sequencing |  |  |
|  |  |  |  |  |  |  |  |  |
| Tiek et al. | 2022 | Nanopore full-length cDNA sequencing | Tumor biopsy | Patient-derived glioblastoma TMZ-sensitive cells and TMZ-resistant in vitro cell line | Yes | Nanopore full-length cDNA sequencing revealed alterations in exon regions and enrichment of splicing mutations in TMZ-resistant cell lines |  |  |
|  |  |  |  |  |  |  |  |  |
| Patel et al. | 2022 | ONT MinION or GridION sequencing | Tumor biopsy | Diffuse glioma | Yes | Created a cost-efficient custom molecular diagnostic workflow using Nanopore, demonstrating its utility in assessing genetical alterations (e.g., MGMT promoter status, methylome, SNVs) |  |  |
|  |  |  |  |  |  |  |  |  |
|  |  |  |  |  |  |  |  |  |
|  |  |  |  |  |  |  |  |  |
| **Study** | **Year** | **TGS method** | **Procedure** | **Type of CNS tumor** | **Compared with other technologies** | **Remarks** |  |  |
| Djirackor et al. | | 2021 | Ultra-low coverage nanopore whole genome sequencing for DNA methylation analysis | Tumor biopsy | Pediatric tumors 16 (sub)types WHO 2016 Adult tumors 13 (sub)types WHO 2016 | Yes | Nanopore-guided DNA methylation classification of CNS tumors can be accurately performed within an intraoperative setting, with a positive impact on surgical strategies and patient outcomes |  |
| Bruzek et al. | 2020 | Nanopore sequencing of ultra-short CSF cf-tDNA fragments | Liquid biopsy | Pediatric high-grade glioma | Yes | The application of Nanopore sequencing for ultra-short pHGG cf-tDNA fragments was found to be highly efficient and sensitive, requiring only small sample quantities. This approach demonstrated a clinical and radiological correlation when assessing the multi-gene molecular response to treatments |  |  |
| Wongsurawat el al. | 2020 | Nanopore Cas9-targeted sequencing (nCATS) | Tumor biopsy | Diffuse glioma | Yes | nCATS provided results within 2 days of surgical resection, accurately identifying IDH1 and IDH2 mutations, MGMT methylation levels, and SNVs |  |  |
|  |  |  |  |  |  |  |  |  |
| Euskirchen et al. | 2017 | Nanopore whole genome sequencing | Tumor biopsy | Gliomas, medulloblastomas and brain metastases (WHO 2016) | Yes | Using nanopore-sequencing allowed for the identification of copy-number alterations, epigenetic modifications and SNVs within one day |  |  |
|  |  |  |  |  |  |  |  |  |
